# Apabetalone and hospitalization for heart failure in patients following an acute coronary syndrome: a prespecified analysis of the BETonMACE study

**DOI:** 10.1186/s12933-020-01199-x

**Published:** 2021-01-07

**Authors:** Stephen J. Nicholls, Gregory G. Schwartz, Kevin A. Buhr, Henry N. Ginsberg, Jan O. Johansson, Kamyar Kalantar-Zadeh, Ewelina Kulikowski, Peter P. Toth, Norman Wong, Michael Sweeney, Kausik K. Ray, the BETonMACE Investigators

**Affiliations:** 1 Monash Cardiovascular Research Centre, Monash University, 246 Clayton Road, Clayton, VIC 3168 Australia; 2 Division of Cardiology, University of Colorado School of Medicine, Aurora, CO USA; 3 Statistical Data Analysis Center, University of Wisconsin-Madison, Madison, WI USA; 4 Irving Institute for Clinical and Translational Research, Columbia University, New York, NY USA; 5 Resverlogix Corporation, Calgary, AB Canada; 6 Division of Nephrology and Hypertension, University of California Irvine, Irvine, USA; 7 CGH Medical Center Sterling, Sterling, IL USA; 8 Cicarrone Center for the Prevention of Cardiovascular Disease, Johns Hopkins University School of Medicine, Baltimore, MD USA; 9 Imperial Centre for Cardiovascular Disease Prevention, Imperial College, London, UK

**Keywords:** BET inhibitors, Acute coronary syndrome, Diabetes, Heart failure, Clinical trial, Cardiovascular disease, Epigenetics, Atherosclerosis

## Abstract

**Background:**

Patients with diabetes and acute coronary syndrome (ACS) are at high risk for subsequent heart failure. Apabetalone is a selective inhibitor of bromodomain and extra-terminal (BET) proteins, epigenetic regulators of gene expression. Preclinical data suggest that apabetalone exerts favorable effects on pathways related to myocardial structure and function and therefore could impact subsequent heart failure events. The effect of apabetalone on heart failure events after an ACS is not currently known.

**Methods:**

The phase 3 BETonMACE trial was a double-blind, randomized comparison of apabetalone versus placebo on the incidence of major adverse cardiovascular events (MACE) in 2425 patients with a recent ACS and diabetes. This prespecified secondary analysis investigated the impact of apabetalone on hospitalization for congestive heart failure, not previously studied.

**Results:**

Patients (age 62 years, 74.4% males, 90% high-intensity statin use, LDL-C 70.3 mg/dL, HDL-C 33.3 mg/dL and HbA1c 7.3%) were followed for an average 26 months. Apabetalone treated patients experienced the nominal finding of a lower rate of first hospitalization for heart failure (2.4% vs. 4.0%, HR 0.59 [95%CI 0.38–0.94], P = 0.03), total number of hospitalizations for heart failure (35 vs. 70, HR 0.47 [95%CI 0.27–0.83], P = 0.01) and the combination of cardiovascular death or hospitalization for heart failure (5.7% vs. 7.8%, HR 0.72 [95%CI 0.53–0.98], P = 0.04).

**Conclusion:**

Apabetalone treatment was associated with fewer hospitalizations for heart failure in patients with type 2 diabetes and recent ACS. Future studies are warranted to define the potential for BET inhibition with apabetalone to prevent heart failure in patients with diabetes and ACS.

## Introduction

Despite current evidence-based treatment, patients with diabetes and acute coronary syndromes (ACS) have a high risk of experiencing subsequent cardiovascular events, including those related to congestive heart failure [1]. Observational studies have reported that congestive heart failure develops in up to 25% of patients who have experienced an ACS within the preceding 12 months, particularly among those with diabetes [2, 3]. The clinical significance of this complication is evidenced by frequent hospitalizations, increased mortality and a substantial increase in the use of health care resources [4–6]. As a result, a therapy that reduces the development of heart failure after ACS would have considerable health and health economic benefits.

Development of heart failure after an ACS may result from ischemic myocardial injury, and also may reflect longer term progression of underlying coronary atherosclerosis and/or diabetic cardiomyopathy [2]. Patients with diabetes comprise about one third of cases of ACS globally and have an elevated risk of heart failure, with or without concurrent coronary disease [7]. Accordingly, there is an unmet need for new therapies that may be initiated following an ACS to prevent heart failure complications in diabetic patients.

Apabetalone is a selective inhibitor of bromodomain and extra-terminal (BET) proteins. Preclinical studies have implicated BET proteins in the regulation of inflammation, calcification, thrombosis, and lipid and lipoprotein metabolism, all of which participate in atherogenesis [8–12]. Each of these pathways have been shown to be modulated by apabetalone [13–21]. In addition, apabetalone may attenuate the development of cardiac hypertrophy [22] and cardiac fibrosis [23]. A recent Phase 3 clinical trial in patients with type 2 diabetes and recent ACS demonstrated a trend towards fewer ischemic cardiovascular events with apabetalone, compared with placebo [24]. The impact of apabetalone on hospitalization for heart failure, however, has not been evaluated in detail.

## Methods

### Study design

The principal results of the BETonMACE study have been described previously [25]. Eligible patients were at least 18 years of age, had an ACS within the preceding 7–90 days, low HDL cholesterol levels, and a diagnosis of type 2 diabetes. Concomitant treatment with high-intensity statin therapy, defined as either atorvastatin 40–80 mg or rosuvastatin 20–40 mg daily, was required. Eligible patients were randomized to treatment with apabetalone 100 mg twice daily or matching placebo. The trial continued until a blinded clinical events committee determined that at least 250 primary events (cardiovascular death, non-fatal myocardial infarction or non-fatal stroke) had occurred. Additional prespecified endpoints measured in the trial included hospitalization for heart failure, including both the first and all subsequent events for each patient.

### Statistical analysis

Baseline characteristics were summarized as percentages for dichotomous data and means (SDs) for approximately normal or medians (IQRs) for non-normal continuous data. Hypothesis test for change in biochemical parameters used ANCOVA models with baseline biomarker value, statin, and country as covariates; results were summarized using least-squares means with 95% confidence intervals. For non-normal data, a Wilcoxon test was used with results summarized using a Hodges–Lehmann pseudo-median and 95% bootstrap confidence intervals. The time-to-event analyses used log-rank tests to calculate P-values and Cox proportional hazards model to estimate the hazard ratio (HR) with 95% confidence interval (CI) and to test for treatment-subgroup interactions. These analyses were stratified by statin and country. Kaplan–Meier curves were also produced. Overall type 1 error control was provided by a sequential gate-keeping procedure that included testing of the primary end point followed by key secondary time-to-event end points. Analyses were performed with SAS software, versions 9.2 or higher (SAS Institute), and R software, version 3.5.1 or higher (R Foundation for Statistical Computing). P-values less than 0.05 were considered statistically significant.

## Results

### Clinical characteristics and medication use

The clinical characteristics of patients are summarized in Table 1. Groups were well balanced by randomization. The median age was 62 years and the majority of patients were male. The index ACS event was myocardial infarction in 75% of patients and unstable angina in 25%. Nearly four in five patients underwent percutaneous coronary intervention for the index ACS, prior to randomization. All patients had a diagnosis of type 2 diabetes and most had additional risk factors such as hypertension and dyslipidemia. Fifteen percent of patients had a prior clinical history of heart failure, before the index ACS. The median time from presentation with ACS to randomization was 38 days in both treatment groups.

**Table 1:** Baseline patient characteristics and medication use

Characteristic	Placebo (N = 1206)	Apabetalone (N = 1212)
Age (years)^a^	62 (56–68)	62 (55–68)
Males (%)	74.0	74.8
Caucasian (%)	87.6	87.7
Body mass index (kg/m^2^)	30.3 ± 5.0	30.2 ± 4.8
Hypertension (%)	87.8	89.4
Dyslipidemia (%)	75.4	74.5
Current or ex-smoker (%)	10.4	12.1
Duration of diabetes (years)	8.7 ± 7.7	84. ± 7.6
Prior myocardial infarction (%)	14.7	14.4
Prior coronary revascularization (%)	21.2	21.4
History of heart failure (%)	14.8	15.1
History of atrial fibrillation (%)	7.2	7.0
History of chronic kidney disease (%)	4.6	5.4
Index ACS event		
STEMI (%)	39.0	38.6
Non-STEMI (%)	35.4	34.2
Unstable angina (%)	25.3	26.7
PCI for index ACS (%)	79.2	79.8
Time from index ACS to randomization (days)^a^	38 (25–62)	38 (25–63.5)
Medications		
Atorvastatin (%)	51.4	51.2
Rosuvastatin (%)	48.6	48.8
High-intensity statin (%)	90.5	89.9
ACE inhibitor/angiotensin II receptor antagonist (%)	92.0	92.3
Beta-blockers (%)	90.2	91.0
Carvedilol, bisoprolol or nebivolol (%)	69.1	67.6
Anti-platelet agents (%)	99.1	98.7
Dual anti-platelet agents (%)	88.3	87.2
Diuretics (%)	53.5	51.3
Mineralocorticoid receptor antagonists (%)	23.1	21.8
Sacubitril/valsartan	0.3	0.1
Diabetes medications		
Metformin (%)	82.0	83.3
Insulin (%)	38.5	36.7
Sulfonlyureas (%)	28.5	30.0
DPP4 inhibitors (%)	14.8	14.9
SGLT2 inhibitors (%)	12.3	12.4
GLP1 receptor agonists (%)	3.7	3.4

Clinical characteristics and medication use of patients treated with apabetalone or placebo. Results expressed as percentage, mean ± standard deviation or ^a^median (interquartile range)

*ACS* acute coronary syndrome, *DPP4* dipeptidyl peptidase 4, *GLP1* glucagon like peptide 1, *PCI* percutaneous coronary intervention, *SGLT2* sodium glucose transporter 2, *STEMI* ST segment myocardial infarction

Concomitant medication use is summarized in Table 1. Established cardioprotective therapies for ACS were widely used in both groups, including high-intensity statin and dual anti-platelet therapy, beta-blockers and inhibitors of the renin–angiotensin–aldosterone system. The average duration of diabetes was 8.5 years. The majority of patients were treated with metformin and more than one third with insulin. The use of anti-diabetic agents demonstrated to have cardiovascular benefit (SGLT2 inhibitors, GLP1 receptor agonists) was relatively low, consistent with reports of slow uptake of these agents in the clinical management of diabetic ACS patients.

### Biochemical parameters

Biochemical parameters and their change after 24 weeks of treatment with placebo and apabetalone are summarized in Table 2. Consistent with the use of high-intensity statin at randomization, the average LDL-C was 70 mg/dL, while the median hsCRP was 2.8 mg/L. Consistent with prior observations, apabetalone-treated patients demonstrated increase in HDL cholesterol and decrease in alkaline phosphatase, compared with placebo. In addition, no significant reduction in estimated glomerular filtration rate was observed in the apabetalone group.

**Table 2:** Biochemistry

Parameter	Placebo	Apabetalone	P value
HbA1c			
Baseline (%)	7.3 (6.4–8.6)	7.4 (6.4–8.7)	
Follow up (%)	7.2 (6.4–8.4)	7.2 (6.4–8.5)	
Absolute change (%)	0.00 [− 0.10, 0.05]	− 0.05 [− 0.15, 0.00]	0.18
Percent change (%)	0.04 [− 0.76, 0.88]	− 0.63 [− 1.49, 0.25]	0.25
LDL-C			
Baseline (mg/dL)	70.9 ± 32.4	69.7 ± 29.8	
Follow up (mg/dL)	63.6 ± 29.4	63.4 ± 28.3	
Absolute change (mg/dL)	− 6.55 [− 8.00, − 5.10]	− 6.34 [− 7.79, − 4.89]	0.37
Percent change (%)	− 0.31 [− 4.13, 3.50]	− 1.57 [− 2.25, 5.38]	0.28
HDL-C			
Baseline (mg/dL)	33.3 ± 5.1	33.3 ± 5.1	
Follow up (mg/dL)	36.6 ± 7.5	38.0 ± 8.0	
Absolute change (mg/dL)	3.34 [2.93, 3.75]	4.75 [4.34, 5.15]	< 0.0001
Percent change (%)	10.9 [9.62, 12.2]	15.4 [14.1, 16.7]	< 0.0001
hsCRP			
Baseline (mg/L)	2.7 (1.1–6.1)	2.9 (1.3–5.9)	
Follow up (mg/L)	1.8 (1.0–4.3)	1.8 (0.9–3.9)	
Absolute change (mg/L)	− 0.53 [− 0.93, − 0.24]	− 0.80 [− 1.3, − 0.44]	0.34
Percent change (%)	− 11.3 [− 20.0, − 1.5]	− 18.4 [− 28.0, − 7.1]	0.16
ALP			
Baseline (U/L)	81.9 ± 34.7	83.3 ± 38.2	
Follow up (U/L)	79.5 ± 29.5	74.3 ± 43.2	
Absolute change (U/L)	− 2.20 [− 3.60, − 0.81]	− 8.98 [− 10.4, − 7.59]	< 0.0001
Percent change (%)	4.22 [0.27, 8.18]	− 8.31 [− 12.2, − 4.37]	< 0.0001
eGFR			
Baseline (mL/min)	102 ± 38.6	105 ± 39.2	
Follow up (mL/min)	105 ± 40.2	106 ± 41.9	
Absolute change (mL/min)	2.02 [0.97, 3.07]	− 0.33 [− 1.38, 1.72]	0.001
Percent change (%)	3.03 [1.87, 4.18]	− 0.46 [− 0.70, 1.61]	0.001

Biochemical parameters at baseline and change after 24 weeks (12 weeks for CRP) of treatment with apabetalone or placebo. Baseline means ± SDs or medians (IQR) are shown. For absolute and percent change, estimates with [95% CIs] are given

*ALP* alkaline phosphatase, *HbA1c* glycated haemoglobin, *HDL-C* high-density lipoprotein cholesterol, *hsCRP* high-sensitivity C-reactive protein (assessed in only a subset of patients), *LDL-C* low-density lipoprotein cholesterol, *eGFR* estimated glomerular filtration rate

### Hospital admissions for heart failure

**Fig. 1:**
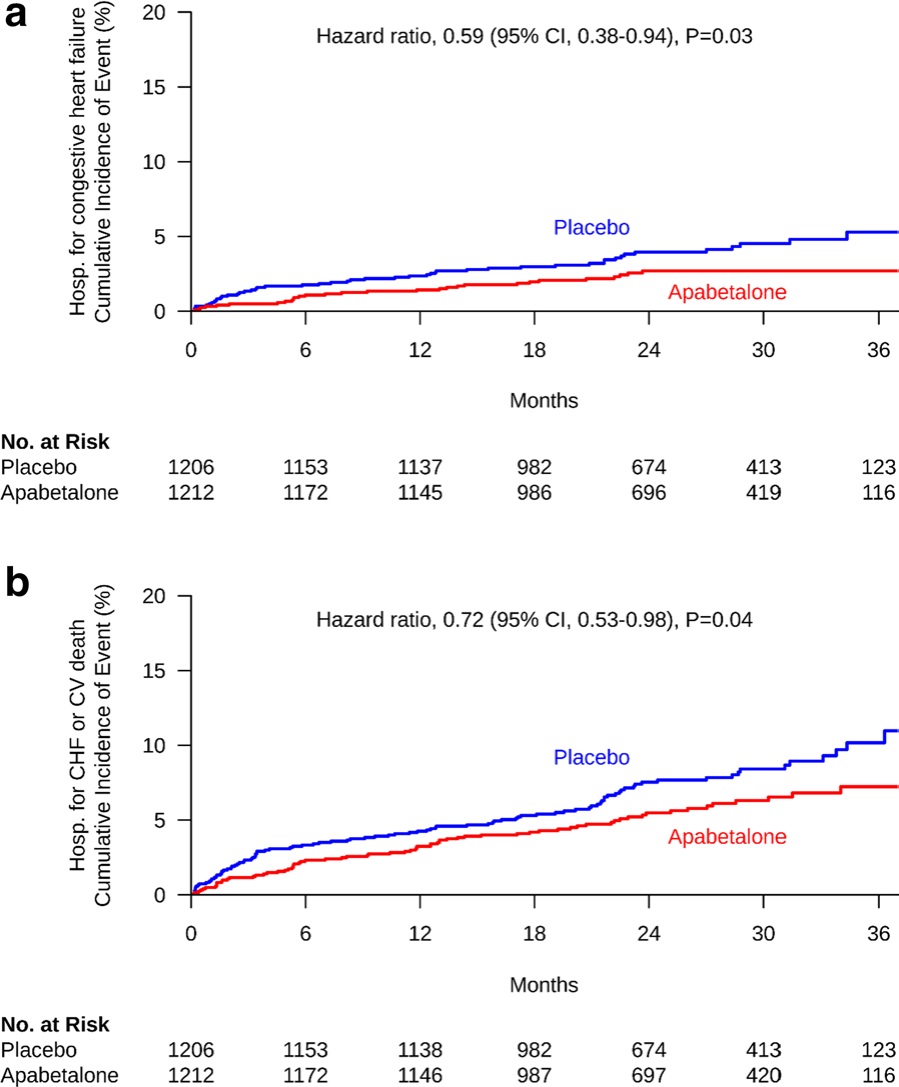
Hospitalization for heart failure. Incidence of first hospital admission for heart failure (**a**) or the combination of first hospital admission for heart failure or cardiovascular death (**b**) in patients treated with apabetalone or placebo

The incidence of cardiovascular events during the trial are summarized in Table 3. Apabetalone-treated patients experienced a lower rate of first [2.4% vs. 4.0%, HR = 0.59 (0.38–0.94), P = 0.03] and total (35 vs. 70, P = 0.01) hospital admissions for heart failure. Of the 105 total hospital admissions for heart failure, 53 (50.5%) occurred in patients with a known history of heart failure prior to study entry (assessed through a general medical history questionnaire rather than a prospective question) and 27 (25.7%) occurred in patients following an acute coronary syndrome during the study. In a *post-hoc* sensitivity analysis of those with no known history of heart failure, the effect of apabetalone on first hospital admissions for heart failure showed a similar hazard ratio, though the overall rates in both treatment arms were lower as might be expected [1.5% vs. 2.6%, HR = 0.56 (0.30–1.05)]. The combination of either first hospital admission for heart failure or cardiovascular death occurred less frequently in apabetalone than placebo-treated patients (5.7% vs. 7.8%, P = 0.04 for all patients, 3.8% vs. 5.7% for those with no known history of heart failure), with qualitative evidence of an early and progressive separation in events (Fig. 1). Since a sequential gate-keeping procedure was used to control overall type 1 error and the primary outcome for BETonMACE was not significant, these P-values should be interpreted with caution.

**Table 3:** Clinical events

Parameter	Placebo (N = 1206)	Apabetalone (N = 1212)	P value
First hospitalization for heart failure, n (%)	48 (4.0)	29 (2.4)	0.03
HR (95% CI)	0.59 (0.38–0.94)		
Total hospitalizations for heart failure, n	70	35	0.01
HR (95% CI)	0.47 (0.27–0.83)		
Cardiovascular death, n (%)	55 (4.6)	45 (3.7)	0.28
HR (95% CI)	0.81 (0.54–1.19)		
First hospitalization for heart failure or cardiovascular death, n (%)	94 (7.8)	69 (5.7)	0.04
HR (95% CI)	0.72 (0.53–0.98)		

Cardiovascular events in patients treated with apabetalone or placebo

**Fig. 2:**
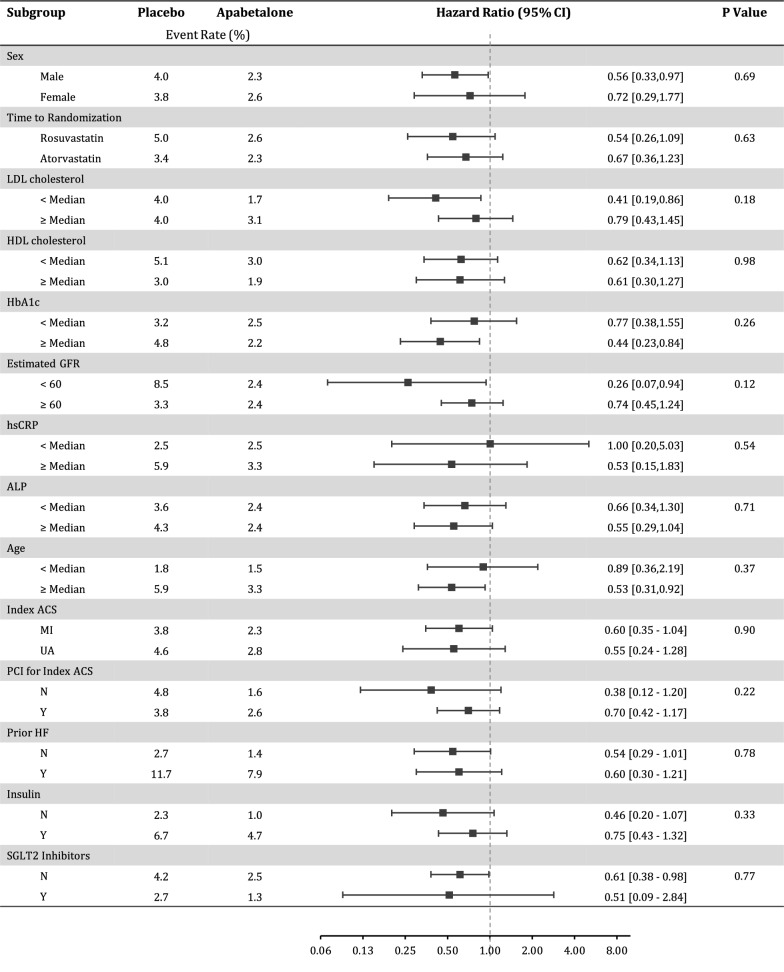
Subgroup analysis for first hospitalization for congestive heart failure. Subgroup analysis of the effect of apabetalone on hospital admissions for heart failure. Interaction P-values are shown. *ACS* acute coronary syndrome, *ALP* alkaline phosphatase, *GFR* glomerular filtration rate, *HbA1c* glycated haemoglobin, *HDL* high-density lipoprotein, *hsCRP* high-sensitivity C-reactive protein, *LDL* low-density lipoprotein, *MI* myocardial infarction, *SGLT2* sodium glucose transporter 2, *UA* unstable angina

Subgroup analysis revealed a consistent effect of apabetalone on hospitalization for heart failure (Fig. 2). Of note, benefit appeared to be independent of sex, lipid profile components, glycemic status, age, and renal function.

## Discussion

The BETonMACE study was the first Phase 3 clinical trial to evaluate the cardiovascular efficacy and safety of a selective BET protein inhibitor, intended to modulate epigenetic factors implicated in cardiovascular disease [26]. The primary analysis of the trial demonstrated a trend towards fewer cardiovascular deaths, non-fatal myocardial infarctions and strokes, although this failed to meet statistical significance [24]. In the current, prespecified analysis, we observed that treatment with apabetalone following an ACS was associated with a lower incidence of hospital admission for heart failure. This finding points to a potentially important clinical benefit of treatment with apabetalone in patients with recent ACS.

The development of heart failure, regardless of the underlying etiology or left ventricular systolic function, typically portends a poor prognosis [4–6]. Such patients enter a deteriorating cycle, characterized by frequent hospital admissions, deteriorating quality of life, an increased risk of death, and substantial health care costs. Randomized controlled trials in patients with chronic heart failure have demonstrated benefit of several interventions such as beta-adrenergic receptor blockade or inhibitors of the renin–angiotensin–aldosterone system [27, 28]. However, there is also a need for therapies that reduce incident heart failure, above and beyond conventional strategies such as blood pressure control or prevention of myocardial infarction with antiplatelet agents or statins. Clinical registries have reported that many patients develop clinically significant heart failure in the first 12 months following an ACS, regardless of the presence of heart failure prior to the index ACS event [2, 3]. When heart failure occurs after ACS, it often requires hospital admission and is associated with an increased risk of death [4–6]. While 15% of patients in BETonMACE reported a prior clinical history of heart failure, the benefit of apabetalone did not appear to be limited to this group. An agent that reduces the risk of incident heart failure would have the potential to improve the prognosis following ACS.

The present observations indicating that apabetalone reduces the risk of hospital admission for heart failure following an ACS is supported by preclinical studies that link BET proteins to a range of mediators of heart failure [8–12]. In those studies, apabetalone favorably modulated immune, oxidative, fibrotic, hypertrophic and vascular calcification pathways implicated in the genesis of both atherosclerosis and heart failure, as evidenced by gene and protein expression profiling in cell studies [13–23]. Other BET inhibitors have also been shown to reduce cardiomyocyte hypertrophy, myocardial fibrosis and apoptosis, in parallel with favorable effects on gene expression [22, 29–32]. These data from BETonMACE suggest that such cellular effects of BET inhibition may translate into clinical benefit for reducing heart failure risk.

Several limitations of the present analysis are recognized. First, neither measures of cardiac function nor circulating biomarkers (e.g., brain natriuretic peptide) associated with heart failure were collected systematically. Such measurements would have identified patients with subclinical cardiac dysfunction, and may have provided insight as to whether or not those patients derived the greatest benefit from apabetalone. Accordingly, we could not make a distinction between heart failure in the setting of preserved or reduced ejection fraction. Second, although hospitalization for heart failure was a prespecified end point in the BETonMACE trial [26], the potential effect of apabetalone on cardiovascular death, myocardial infarction, or stroke, the primary endpoint of the trial, failed to meet statistical significance [24]. As a result, the observation of a favorable effect on a secondary endpoint should be considered an exploratory finding. Third, the study was performed exclusively in patients with type 2 diabetes, a group of patients known to be at high risk of both recurrent ischemic events after ACS and heart failure per se. Whether similar findings would be observed in a broader ACS cohort is unknown. Fourth, the benefit of apabetalone on heart failure events was observed on a background of high utilization of beta-blockers and renin-angiotensin system inhibitors, agents demonstrated to be beneficial in heart failure [27, 28]. However, there was little background use of SGLT2 inhibitors, another class reported to reduce heart failure events [33, 34], although potential efficacy in the post ACS setting has not been established. Fifth, a prior history of heart failure was collected by voluntary patient reporting, limiting the ability to investigate the impact of apabetalone in patients with established heart failure in the current analysis. Finally, given that the primary endpoint of the BETonMACE study did not meet statistical significance, the current findings should be considered nominal.

## Conclusion

In summary, use of the selective BET inhibitor, apabetalone was associated with a lower rate of hospital admissions for heart failure in patients with type 2 diabetes and recent ACS. The mechanism underlying this potential benefit remains uncertain and requires further investigation. If this finding is replicated in future clinical trials, it may provide an important new therapeutic strategy to modulate the high rate of both atherothrombotic and heart failure-related complications following an ACS in patients with diabetes.

## Data Availability

The data that support the findings of this study are available from Resverlogix but restrictions apply to the availability of these data, which were used under license for the current study, and so are not publicly available. Data are however available from the authors upon reasonable request and with permission of Resverlogix.

## Abbreviations

ACS: Acute coronary syndrome
BET: Bromodomain and extra-terminal
CI: Confidence interval
GLP-1: Glucagon like peptide-1
HbA1c: Glycated hemoglobin
HDL-C: High-density lipoprotein cholesterol
HR: Hazard ratio
hsCRP: High sensitivity C-reactive protein
LDL-C: Low-density lipoprotein cholesterol
MACE: Major adverse cardiovascular events
SGLT2: Sodium glucose transporter 2
